# A Validation-Driven Explainable Deep Ensemble Framework for Image-Based Saffron Adulteration Detection

**DOI:** 10.3390/foods15101661

**Published:** 2026-05-10

**Authors:** Syed Nisar Hussain Bukhari, Kingsley A. Ogudo

**Affiliations:** 1 National Institute of Electronics and Information Technology (NIELIT) J&K, Srinagar 191132, India; 2 Department of Electrical & Electronic Engineering Technology, University of Johannesburg, Johannesburg 2094, South Africa; kingsleyo@uj.ac.za

**Keywords:** saffron adulteration detection, deep ensemble learning, convolutional neural networks, validation-driven learning, explainable artificial intelligence, image-based authentication, *Crocus sativus* L., transfer learning

## Abstract

Saffron (*Crocus sativus* L.), one of the world’s most valuable spices, is highly vulnerable to adulteration due to its premium market price and the limitations of conventional analytical methods for rapid, non-destructive authentication. Although recent deep learning-based approaches have reported promising accuracy, many rely on single models or naïve ensembles and lack rigorous validation and statistical reliability assessment. This study proposes a validation-driven and explainable deep ensemble framework for image-based saffron adulteration detection. Multiple pretrained convolutional neural networks (DenseNet169, ResNet50, and VGG16) are integrated using a validation-driven weighted ensemble strategy in which fusion weights are computed exclusively from validation performance within the training folds and fixed prior to evaluation on the held-out fold, thereby preventing information leakage between model selection and performance assessment. The proposed framework achieved 98.61% classification accuracy, 98.17% F1-score, and 98.61% AUC, outperforming the best individual base model by up to 1.4% in F1-score. Stratified five-fold cross-validation demonstrated stable performance, with a mean accuracy of 97.81% ± 0.53, confirming robustness across data splits. Statistical validation using McNemar’s test (*p* < 0.05) and 5 × 2 cross-validated significance testing verified that performance improvements over constituent models are statistically reliable. Grad-CAM-based explainability and background-invariance analysis further demonstrated that predictions are driven primarily by intrinsic filament-level characteristics, with only a marginal (~0.9%) performance reduction under ROI-cropped evaluation. The proposed framework provides a robust, interpretable, and statistically validated solution for saffron authentication and offers methodological insights for reliable image-based food adulteration detection under limited data conditions.

## 1. Introduction

Saffron (*Crocus sativus* L.) is among the most expensive and highly prized spices worldwide, valued for its intense color, distinctive aroma, and unique flavor profile [1]. The spice is derived from the dried stigmas of this sterile triploid plant, and its cultivation requires labor-intensive manual harvesting, resulting in low yield and high market value [2]. Major saffron-producing regions include Iran, India, Spain, and Morocco [3], with Iran contributing nearly 90% of global production [4]. India ranks second, with cultivation primarily concentrated in the Pampore region of Jammu and Kashmir [5]. Kashmiri saffron, locally referred to as Koung and commonly known as Kesar in India, is particularly prized for its thicker stigmas, deep crimson coloration, and strong aroma, attributes that contribute to its distinct identity among globally cultivated saffron varieties [6].

**Figure 1:**
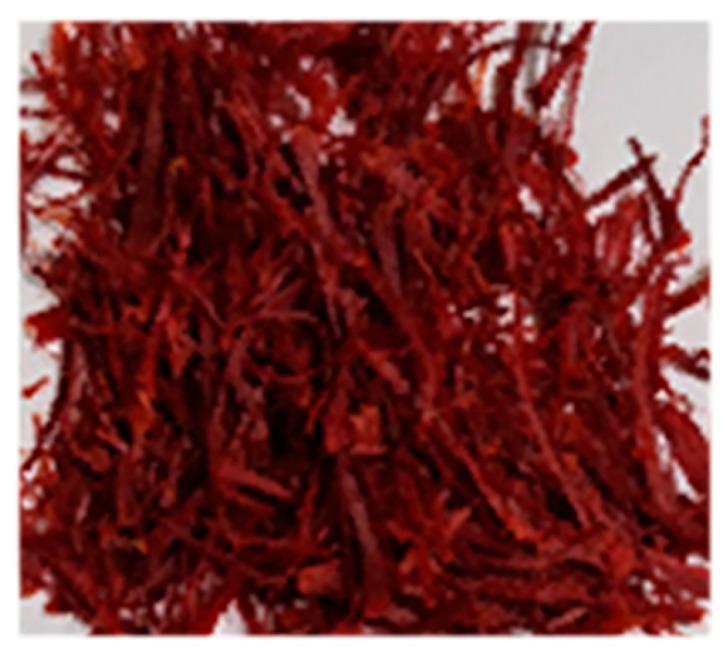
Pure Saffron.

**Figure 2:**
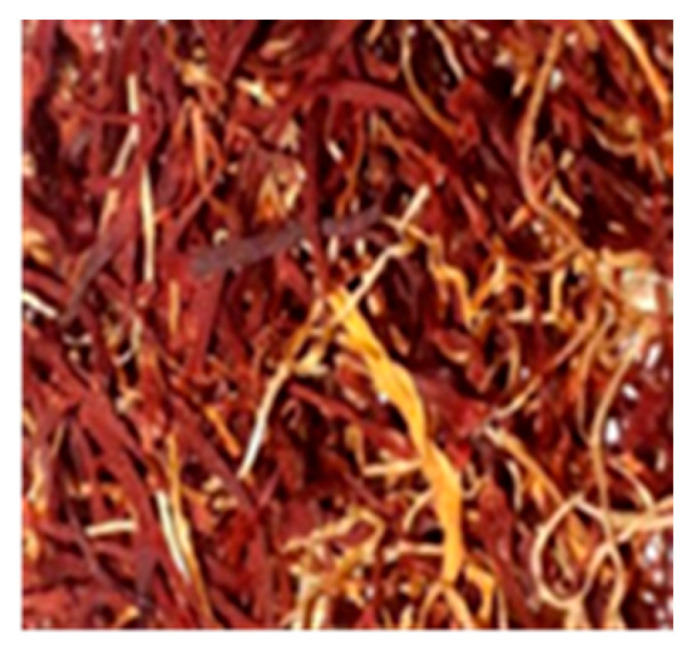
Adulterated Saffron.

The high market value of saffron has made it increasingly susceptible to adulteration through practices such as dyeing foreign plant material, blending synthetic fibers, or incorporating visually similar substitutes, including safflower and corn silk [3,7]. Such adulteration compromises product authenticity, undermines consumer trust, and poses economic risks to genuine producers. Representative examples of authentic and adulterated saffron samples used in this study are shown in Figure 1 and Figure 2. Authentic saffron is typically characterized by deep red, unbroken stigmas with naturally tapering ends, slight curvature, and a strong characteristic aroma [7]. In contrast, adulterated samples often contain broken threads, artificial coloration, and extraneous plant materials or synthetic filaments, which deviate markedly in texture, color uniformity, and olfactory properties.

Despite the seriousness of the issue, reliable detection of saffron adulteration remains a persistent challenge, particularly in Kashmir, where such practices are widespread [8]. Traditional methods such as manual inspection, microscopy, or chemical analysis are not only time-consuming and labor-intensive but also require expert supervision and are prone to subjectivity and error. To address these challenges, several analytical approaches have been explored. Chromatographic methods such as thin-layer chromatography (TLC) [9], gas chromatography (GC) [10], and liquid chromatography–mass spectrometry (LC-MS) [11] offer high sensitivity and specificity but are generally expensive and require specialized equipment and skilled personnel. Molecular techniques, including polymerase chain reaction (PCR) [12] and nuclear magnetic resonance (NMR) spectroscopy [13], have also been employed with promising results [14]. However, these techniques are rarely scalable or suitable for rapid, real-time detection in field conditions.

Recent studies have explored non-invasive and rapid methods for saffron authentication. Optical techniques such as UV–visible spectroscopy [15] and near-infrared (NIR) spectroscopy [16] have been used for classification and compositional analysis across different regions. NIR combined with chemometric methods has also been applied to detect common adulterants such as lotus stamens and corn stigmas [17,18,19]. While these approaches are non-destructive, their performance can be affected by variations in lighting and sample conditions. Microscopy- and chromatography-based methods, including TLC and HPLC, have also been used to distinguish genuine saffron from adulterants based on chemical and visual characteristics [20].

In parallel, image-based machine learning (ML) and deep learning (DL) techniques have emerged as scalable alternatives. Early approaches utilized artificial neural networks and sensor-based systems for adulteration detection [21,22]. Hyperspectral imaging and spectral analysis have further improved classification performance in controlled settings [23]. Machine vision techniques have also been employed for saffron grading, drying analysis, and regional classification using color and texture features [24,25,26,27]. More recently, convolutional neural networks (CNNs) have demonstrated strong potential for saffron adulteration detection, with studies evaluating multiple architectures and deep models for distinguishing pure and adulterated samples [28,29].

Despite these advances, many existing methods rely on handcrafted features or hardware-intensive systems that limit scalability and practical deployment. Although DL-based image analysis shows promise, its systematic application to Kashmiri saffron remains limited, particularly within rigorously validated ensemble frameworks. Existing CNN-based studies often focus on single-model performance or employ simple ensemble strategies without clearly separating validation and evaluation stages, increasing the risk of optimistic performance estimates. Moreover, evaluation is typically centered on accuracy, with limited attention to statistical reliability, robustness to background variations, or consistent interpretability across adulterant types. To address these limitations, this study proposes a transfer learning-based deep ensemble learning approach for image-based saffron adulteration detection in Kashmiri saffron. Widely used CNN architectures such as DenseNet169, ResNet50, and VGG16 are employed due to their complementary strengths. DenseNet facilitates efficient feature reuse and is effective in capturing fine-grained texture patterns, ResNet enables stable deep feature learning through residual connections, and VGG provides consistent hierarchical feature extraction. The combination of these architectures allows the proposed framework to capture diverse visual characteristics relevant to saffron adulteration detection. Model behavior is further examined through stratified cross-validation, statistical significance testing, robustness analysis, and explainable visual interpretation.

Accordingly, the study investigates the following research questions:

Q1: Can DL-based methods reliably distinguish authentic saffron from adulterated saffron using RGB image data under limited sample conditions?

Q2: How do different transfer learning-based CNN architectures contribute to saffron adulteration detection when integrated within a validation-aware ensemble framework?

Q3: Are the performance gains achieved by the proposed ensemble statistically reliable across cross-validation folds compared with individual CNN models?

Q4: To what extent are the model’s predictions robust to background variations and resistant to shortcut learning arising from acquisition-specific cues?

Q5: Which visual regions and feature representations most strongly influence adulteration-related decisions, and how do individual model components contribute to overall ensemble performance?

By addressing these questions, this study moves beyond purely accuracy-oriented evaluation and instead emphasizes reliability, robustness, and interpretability in deep learning-based saffron adulteration detection. The proposed framework integrates multiple pretrained CNN backbones using a validation-driven weighted ensemble strategy to capture complementary visual representations while reducing sensitivity to subtle adulteration patterns and intra-class variability. The evaluation further incorporates statistical significance testing, robustness analysis through background-invariance experiments, and explainable visual interpretation to examine model behavior. Together, these components provide a more reliable and interpretable framework for saffron authentication and offer methodological insights for image-based food adulteration detection under limited data conditions.

The main contributions of this work are summarized as follows:

1. A deep ensemble approach is proposed in which fusion weights are computed exclusively from training-fold validation performance and fixed prior to evaluation, mitigating information leakage and optimistic bias commonly overlooked in ensemble-based food authentication studies.
2. A cross-validation-aware assessment protocol is employed, combining stratified five-fold cross-validation with corrected McNemar significance testing to establish the statistical reliability of performance improvements beyond accuracy reporting.
3. Background-invariance experiments using region-of-interest cropped images demonstrate that the proposed model relies primarily on intrinsic saffron filament features rather than spurious background or acquisition-specific cues.
4. Grad-CAM-based visual explanations and per-adulterant performance analysis provide insights into the decision behavior of the model across visually similar adulterants, highlighting both strengths and inherent limitations relevant to real-world deployment.

## 2. Materials and Methods

This section describes the experimental pipeline adopted in this study, including the dataset preparation, deep learning architectures employed, transfer learning strategy and ensemble construction.

### 2.1. Convolutional Neural Networks (CNNs)

Convolutional neural networks are a well-established class of DL models widely used for image analysis due to their ability to learn hierarchical spatial representations directly from raw pixel data [30]. The CNN backbones employed in this work were selected to provide complementary feature representations for saffron image classification, balancing fine-grained texture sensitivity, structural abstraction, and training stability [31].

The selection of DenseNet169, ResNet50, and VGG16, which are explained next, was guided by their complementary architectural characteristics and their established performance in transfer learning-based image classification tasks. DenseNet architectures are known for effective feature reuse and fine-grained texture learning, ResNet architectures provide strong structural representation through residual connections, while VGG networks offer stable hierarchical feature extraction with relatively simple architecture. The objective of this work was not to perform an exhaustive architectural search but rather to evaluate how complementary CNN backbones can be combined within a validation-driven ensemble framework to improve reliability and robustness in saffron adulteration detection. These models were therefore selected as representative architectures with differing connectivity patterns and feature-learning behaviors.

### 2.2. DenseNet169

In the proposed framework, DenseNet169 serves as a primary feature extractor for capturing fine-grained filament textures and subtle color variations that are characteristic of authentic saffron stigmas [32]. Such characteristics are particularly relevant for saffron adulteration detection, where subtle differences in filament texture, thickness, and color gradients play a critical role. The dense feature propagation also contributes to stable gradient flow during fine-tuning, making DenseNet169 well suited for limited-data scenarios.

### 2.3. ResNet50

ResNet50 is included to capture mid-level and structural representations through residual learning. Its skip connections enable effective optimization while preserving discriminative information across network depth [33]. Within the ensemble framework, ResNet50 contributes complementary structural representations that help capture variations in filament orientation and morphology across authentic and adulterated samples.

### 2.4. VGG16

VGG16 is incorporated for its sequential architecture and consistent use of small convolutional filters, which facilitate hierarchical feature extraction. Despite its relatively simple design, VGG16 has demonstrated strong generalization capabilities in visual classification tasks [34]. In the proposed ensemble approach, VGG16 provides stable hierarchical visual features that complement the deeper architectures, enabling the ensemble to capture both low-level texture information and higher-level structural characteristics.

### 2.5. Transfer Learning Strategy

Transfer learning is employed to address the limited availability of labeled saffron images by leveraging feature representations learned from large-scale natural image datasets. All CNN backbones are initialized with ImageNet-pretrained weights, and the classification layers are replaced with task-specific fully connected layers [35].

During training, a partial fine-tuning strategy was applied. For VGG16, convolutional blocks 1–3 were kept frozen, while blocks 4–5 were fine-tuned. For ResNet50, layers up to conv3_x were frozen, while conv4_x and conv5_x were fine-tuned. For DenseNet169, dense blocks 1–2 were frozen, while dense blocks 3–4 were fine-tuned.

The models were trained for 50 epochs using the Adam optimizer with a learning rate of 0.002 (β_1_ = 0.9, β_2_ = 0.999, ε = 1 × 10^−7^). Early stopping (patience = 10) and learning rate scheduling (ReduceLROnPlateau) were applied based on validation loss. The same configuration was used across all models and cross-validation folds to ensure reproducibility.

### 2.6. Validation-Aware Ensemble Learning

Ensemble learning is a powerful machine learning paradigm that combines the predictions of multiple models to improve overall performance, reduce generalization error, and increase robustness. By aggregating diverse learners, ensemble methods often outperform individual models, particularly in complex image classification tasks involving subtle inter-class variations. Each model contributes to the ensemble by leveraging its unique feature extraction capabilities [36]. In the proposed approach, all base models are independently fine-tuned and trained on the same dataset under identical training and augmentation settings [37]. For a given test sample, each fine-tuned model predicts posterior class probabilities. Rather than employing a simple equal-weight averaging scheme, the final ensemble prediction is obtained using a validation-driven weighted averaging strategy, where each model contributes proportionally according to its validation performance. For a classification problem with m base models and n classes, let *Pk*∈ℝ*n* denote the probability prediction vector produced by the *k*th model. The ensemble prediction is computed as:

$$P_{ensemble}=\sum_{k=1}^{m}w_{k}P_{k}$$

where *wk* represents the ensemble weight assigned to the *kth* model and satisfies the constraint $\sum_{k=1}^{m}w_{k}=1$.

The ensemble fusion weights shown in Table 1 were computed exclusively using validation performance obtained from the training folds during each cross-validation iteration. Specifically, for a given fold, the base models were trained using the training subset and their performance is evaluated only on the corresponding validation subset within the same training partition. The resulting validation scores were then used to determine the ensemble weights, which were fixed before evaluating the model on the held-out fold. At no stage was the held-out test fold used for training, hyperparameter tuning, or ensemble weight estimation. All model selection and optimization steps were performed exclusively using training and validation data. This strict separation ensured that ensemble construction remained independent of the evaluation data and prevented potential information leakage.

DenseNet169 consistently received the highest ensemble weight across folds, indicating its superior ability to capture fine-grained textural and morphological features of saffron filaments. ResNet50 and VGG16 contributed comparably, providing complementary mid-level and structural representations. The relatively small variation in ensemble weights across folds suggests that the estimated weights remained broadly consistent across different training splits.

**Figure 3:**
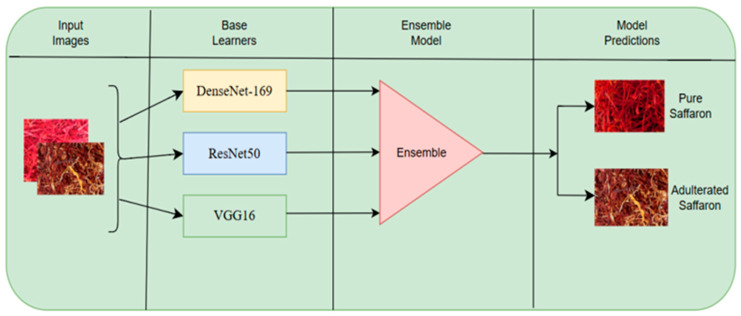
Proposed deep ensemble approach.

Although multiple base learners were combined, bootstrap resampling was not employed. Therefore, the proposed method is more accurately characterized as a validation-driven weighted averaging ensemble rather than classical bagging. This fusion strategy allows stronger base learners to contribute more significantly to the final decision while preserving the complementary strengths of all models. The overall ensemble architecture of the proposed approach is illustrated in Figure 3. The final predicted class label is determined as the class with the highest posterior probability from P_ensemble_.

### 2.7. Experimental Setup

The experimental setup was meticulously designed to ensure optimal performance and accurate evaluation of the proposed model. The implementation was carried out using Keras, an open-source DL library that interfaces seamlessly with TensorFlow. TensorFlow’s backend enables hardware-level optimization through GPU acceleration, allowing Keras to streamline the construction, training, and fine-tuning of DL models with enhanced computational efficiency. The hardware configuration utilized for this study comprised an Intel Core i7 3770 CPU, 8 GB Dual-Channel DDR3 memory, a 4 GB NVIDIA GeForce GTX 960 GPU, and a 240 GB Kingston SSD. These specifications provided adequate computational resources to train and evaluate the models effectively. The DL models employed in this study, namely DenseNet169 [32], ResNet50 [33], and VGG16 [34], were initialized with ImageNet weights by setting include_top = false, allowing the models to extract domain-specific features from raw image data. In the dense layers, “ReLU” activation was used for non-linear transformation, and the “he_normal” initializer was employed for kernel weight initialization. A dropout rate of 0.3 was applied to each dense layer to reduce the risk of overfitting. The output layers used the softmax function to classify images into their respective categories. The models were fine-tuned using the Adam optimizer with an initial learning rate of 0.002, β_1_ = 0.9, β_2_ = 0.999, and ε = 1 × 10^−7^. The same fine-tuning configuration and training parameters were used consistently across all folds and base models. The binary cross-entropy loss function was utilized, with accuracy chosen as the evaluation metric, and the stratified five-fold cross-validation protocol was employed.

The dataset was partitioned into five mutually exclusive folds while preserving class proportions. In each cross-validation iteration, one fold was reserved as the test fold for final evaluation, while the remaining four folds formed the training set. A small validation subset was then derived from the training folds and used exclusively for monitoring training and estimating ensemble fusion weights. The test fold was never used during model training or weight estimation, ensuring a strict separation between model selection and final performance evaluation. Each architecture was trained for 50 epochs using the fit_generator method. Additionally, data augmentation techniques, such as random rotations, flips, zooms, and brightness adjustments, were applied exclusively to the training data within each fold to increase variability during model learning. Early stopping was applied to prevent overfitting by monitoring the validation loss during training. Training was terminated if the validation loss did not improve for 10 consecutive epochs. In addition, learning rate scheduling was implemented using the ReduceLROnPlateau strategy, which reduced the learning rate when validation performance plateaued. Finally, the validation-driven weighted averaging ensemble was employed [38] wherein predictions from the fine-tuned DenseNet169, ResNet50, and VGG16 models were aggregated to compute the final classification output. This approach leveraged the complementary strengths of each model, improving overall stability and accuracy.

### 2.8. Dataset Description

For this study, a comprehensive dataset was curated to ensure the effective training and evaluation of the proposed models. The dataset consisted of high-resolution images obtained from reliable sources to maintain authenticity and relevance to the classification task. The dataset comprises a total of 1080 images, including both authentic and adulterated saffron samples. Among these, 560 samples are classified as authentic, while 520 samples represent various adulterated forms. The saffron samples used in this study were obtained from the Pampore region of Jammu and Kashmir, India, which is widely recognized as the primary cultivation area of Kashmiri saffron (*Crocus sativus* L.). Samples were collected from multiple sources, including local saffron growers, individuals cultivating saffron under regional agricultural projects, and local shopkeepers involved in saffron trade. Authentic saffron samples were obtained directly from growers to ensure their provenance and quality. Adulterated samples consisted of plant materials commonly reported as saffron substitutes, including male parts of saffron flowers, maize coverings, and safflower petals. The authenticity of saffron samples was verified based on their source and characteristic morphological features, including the deep red color of the stigmas, trumpet-shaped filament structure, and typical aroma associated with genuine saffron. These features are widely recognized indicators used in saffron quality assessment. The collected samples were subsequently photographed under controlled acquisition conditions to create the image dataset used in this study. Representative images from both authentic and adulterated samples are displayed in Figure 1 and Figure 2. To ensure balanced representation, the dataset was categorized according to the type of adulterant. The composition is detailed in Table 2.

Although the dataset includes several visually distinct adulterant types (male saffron parts, maize coverings, and safflower petals), the classification task in this study is formulated as a binary authentication problem. Specifically, the model predicts whether a sample corresponds to pure saffron or adulterated saffron. Therefore, all adulterant categories are grouped into a single adulterated class during model training. This formulation reflects practical authentication scenarios where the primary objective is to determine whether saffron is genuine or adulterated.

Image acquisition was conducted using a Sony Alpha ILCE-5100 L camera and a Samsung Galaxy M53 128 GB smartphone. Images were captured under diverse conditions, including environmental noise and distortions, and under both natural and indoor lighting conditions to reflect realistic acquisition scenarios while enhancing dataset robustness and improving the models’ generalization capability. The imaging devices were positioned at a distance of 13 cm from the samples. During the acquisition process, the following camera settings were employed:

Shutter speed: 1/2400 s;

Exposure time: 10 s (without flash);

Aperture range: f/1.5 to f/2.4;

Lens focal length: 26 mm;

ISO sensitivity: Between 50 and 800.

Images were captured at their maximum resolution of 3024 × 3024 pixels and stored in JPG format for consistency and efficient storage. As part of the preprocessing step, each image was manually labeled based on the known origin of the sample and visual inspection of morphological characteristics. The labeling process categorized images into pure saffron or adulterated saffron classes prior to model training.

Since color characteristics represent an important discriminative attribute in saffron authentication, additional preprocessing steps were considered to reduce the effect of device-dependent color variations and illumination differences. All images were normalized during preprocessing to standardize pixel intensity ranges, and data augmentation techniques involving controlled brightness variations were applied during training to improve robustness to illumination changes. These measures helped reduce the influence of minor variations in color rendering across different acquisition devices and lighting conditions while preserving the intrinsic color characteristics of saffron filaments.

### 2.9. Evaluation Metrics

The following standard classification metrics were used to quantitatively evaluate model performance across cross-validation folds. These metrics are derived from the confusion matrix elements, namely true positives (TPs), true negatives (TNs), false positives (FPs), and false negatives (FNs) [39]. TPs and TNs represent correct predictions, while FPs and FNs correspond to misclassifications.

The precision metric, defined in Equation (1), calculates the proportion of correctly predicted positive cases out of all predicted positive cases.

(1) $$Precision=\frac{TP}{TP+FP}$$

Accuracy, as expressed in Equation (2), measures the overall correctness of the model by calculating the proportion of accurate predictions (both positive and negative) to the total predictions made.

(2) $$Accuracy=\frac{TP+TN}{TP+FP+TN+FN}$$

Recall, or sensitivity, shown in Equation (3), assesses the model’s ability to correctly identify true positive cases.

(3) $$Recall=\frac{TP}{TP+FN}$$

To provide a balanced evaluation of precision and recall, the F1-score is utilized, as described in Equation (4). This metric represents the harmonic mean of precision and recall, offering a comprehensive view of the model’s classification performance by considering both types of misclassifications.

(4) $$F1\_Score=\frac{2\cdot (Precision\cdot Recall)}{Precision+Recall}$$

The area under the receiver operating characteristic curve (AUROC), as shown in Equation (5), evaluates the model’s ability to distinguish between the positive and negative classes across all possible thresholds.

(5) $$AUROC=\int_{0}^{1}TPR(FPR)d(FPR)$$

The AUROC values are computed for each fold using the predicted class probabilities, and the final reported value represents the mean AUROC obtained across all cross-validation folds. All reported performance values for the evaluation metrics correspond to the mean and standard deviation computed across the five cross-validation folds.

### 2.10. Implementation

The implementation phase of this study was designed to optimize the performance of the proposed ensemble model for saffron adulteration detection. The three selected TL models, namely DenseNet169, ResNet50 and VGG16, were fine-tuned using pre-trained ImageNet weights, with modifications to their output layers to suit the specific classification task. The models were initialized with the “include_top = False” setting to extract high-level features from input images and were augmented with custom fully connected layers employing the ReLU activation function, He normal kernel initializations, and a softmax output layer. The input images were resized to 224 × 224 pixels to ensure compatibility with the selected architectures. Advanced image pre-processing techniques were employed, including normalization to scale pixel values between 0 and 1 and data augmentation techniques like random rotations, flips, and zooms to mitigate overfitting and improve generalizability. During training, batches of images were fed into the CNN models, where the convolutional layers automatically extracted hierarchical visual features related to saffron filament morphology, texture, and color characteristics. The resulting feature representations were subsequently processed by the fully connected layers to produce the final classification output, indicating whether the sample corresponded to pure or adulterated saffron. Algorithm 1 outlines the steps involved in the implementation of the scheme.
**Algorithm 1:** Deep Ensemble Learning Approach for Image-based Saffron Adulteration Detection**  Inputs:**  𝒮: Saffron image dataset  𝒪: Optimizer = Adam  η: Learning Rate = 0.002  μ: Dropout Rate = 0.3  ℬ: Batch Size = 16  E: Number of Epochs = 50  ℱ = {F_1_, F_2_, F_3_}: Set of Fine-tuned Models = [DenseNet169, ResNet50, VGG16]  θ: Pre-trained Weights  **Output:**  Final predicted class label for each test image    **for** each model Fᵢ ∈ ℱ **do**            Initialize training and validation sets specific to Fᵢ            Load pre-trained weights θ into Fᵢ                **while** epoch < E **do**                        Perform forward pass and compute categorical cross-entropy loss on training set                        Backpropagate gradients and update weights using optimizer 𝒪                **end while**             Save prediction probability scores from Fᵢ on validation set    **end for**
    **for** each image x ∈ test set **do**                Aggregate prediction scores from all models in ℱ                Compute final score = average of individual model scores                Assign predicted class = class with maximum probability in final score        **end for**

Each model was trained for 50 epochs using the Adam optimizer, with an initial learning rate of 0.002. To enhance convergence, learning rate scheduling was applied using the ReduceLROnPlateau callback, which reduced the learning rate when the validation loss plateaued. Early stopping was also employed to prevent overfitting by halting training when no significant improvement in validation performance was observed for 10 consecutive epochs. Key hyperparameters used during training, such as batch size, learning rate, and dropout rates, are summarized in Table 3. The hyperparameters reported in Table 3 were selected based on preliminary validation experiments aimed at achieving stable convergence while minimizing overfitting on the relatively small dataset. In particular, smaller batch sizes were observed to improve generalization by introducing greater stochasticity during gradient updates. A batch size of 4 was therefore adopted, as it provided the most stable training behavior and consistent validation performance within the available GPU memory constraints. The final set of hyperparameters was chosen after evaluating multiple configurations on the validation subset derived from the training folds, ensuring that the selected settings provided reliable performance without overfitting.

The ensemble strategy utilized the probability prediction matrices generated by the individual models. These matrices were combined using a weighted averaging approach, assigning optimal weights to each model based on their validation performance. The ensemble method effectively captured diverse feature representations learned by each model, improving overall classification accuracy. The combined prediction scores were used to determine the final classifications for the test samples.

## 3. Results

This section presents the experimental results obtained from the proposed deep ensemble framework and the individual base CNN models. The evaluation focuses on the classification performance achieved under the stratified five-fold cross-validation protocol described in the methodology. Performance is assessed using multiple evaluation metrics, including accuracy, sensitivity, specificity, precision, F1-score, and area under the receiver operating characteristic curve (AUROC).

**Figure 4:**
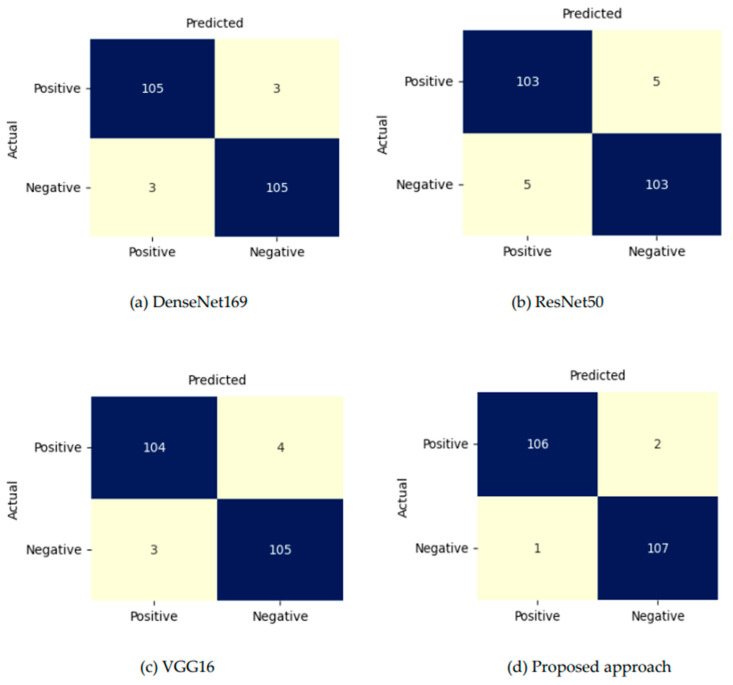
Confusion matrices of the predictions by base models and the proposed approach: (**a**) DenseNet169, (**b**) ResNet50, (**c**) VGG16, (**d**) proposed approach.

**Figure 5:**
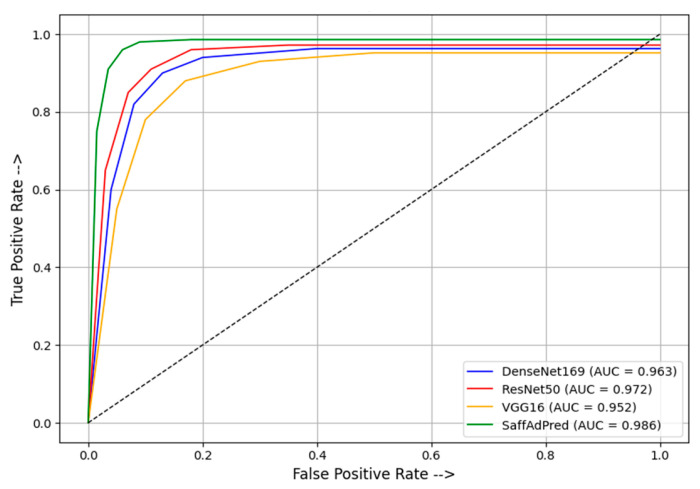
ROC curves of the base models and the proposed approach, named Deep-SADet in the legend.

**Figure 6:**
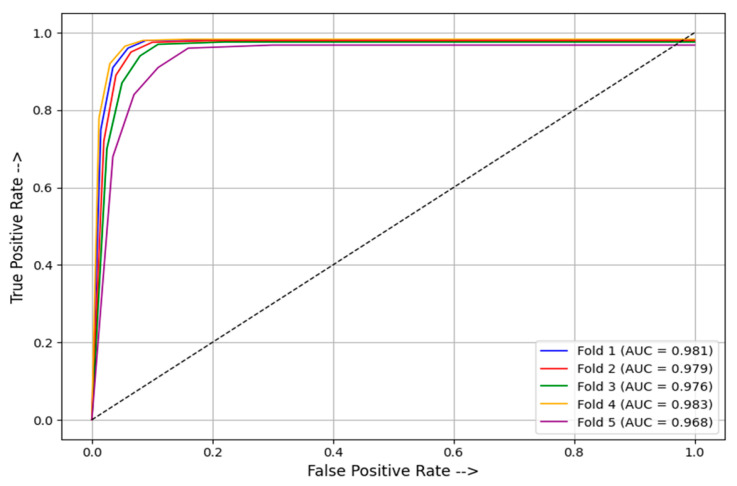
ROC curves of the proposed approach obtained using 5FCV.

The performance of individual models and the proposed model is presented in Table 4, while the overall results using five-fold cross validation (5FCV) are summarized in Table 5. Performance metrics were computed separately for each fold using the corresponding test subset and subsequently averaged across all folds to obtain the final reported values. The confusion matrices for the test set are depicted in Figure 4, and the receiver operating characteristic (ROC) curves for individual models and the proposed model, as depicted in Figure 5, illustrate the classification effectiveness and reliability of the proposed model. The ROC curves of the proposed model using 5FCV are shown in Figure 6, clearly demonstrating that proposed model is effective in distinguishing between pure and adulterated saffron image samples.

It is evident from Table 4 that the proposed approach, which combines multiple pre-trained CNN architectures—namely DenseNet169, ResNet50 and VGG16—to leverage complementary feature representations, has achieved a significantly improved classification accuracy of 98.61%, alongside superior sensitivity (98.65%) and specificity (98.14%), thereby demonstrating its ability to correctly identify both adulterated and non-adulterated samples with minimal false classifications. It maintained a high precision of 98.03%, indicating reliable positive predictions, while its F1-score of 98.17% confirmed a balanced performance between precision and recall. Additionally, the model’s AUC score of 98.61% reflects strong discriminative capability across decision thresholds. In contrast, the other models exhibited relatively lower and less consistent performance across these metrics, with DenseNet169 achieving the second-best accuracy (97.21%) and ResNet50 showing a slightly better AUC (97.24%) despite lower accuracy. Unlike the base models, which often exhibited trade-offs between sensitivity and specificity, the proposed approach maintained an optimal balance, suggesting excellent generalization to unseen data and robustness against class imbalance. The close alignment between its precision and recall metrics further confirms the model’s stable behavior under varying input conditions. These characteristics can be attributed to its domain-specific architectural design, optimized to capture subtle visual features in saffron images.

**Figure 7:**
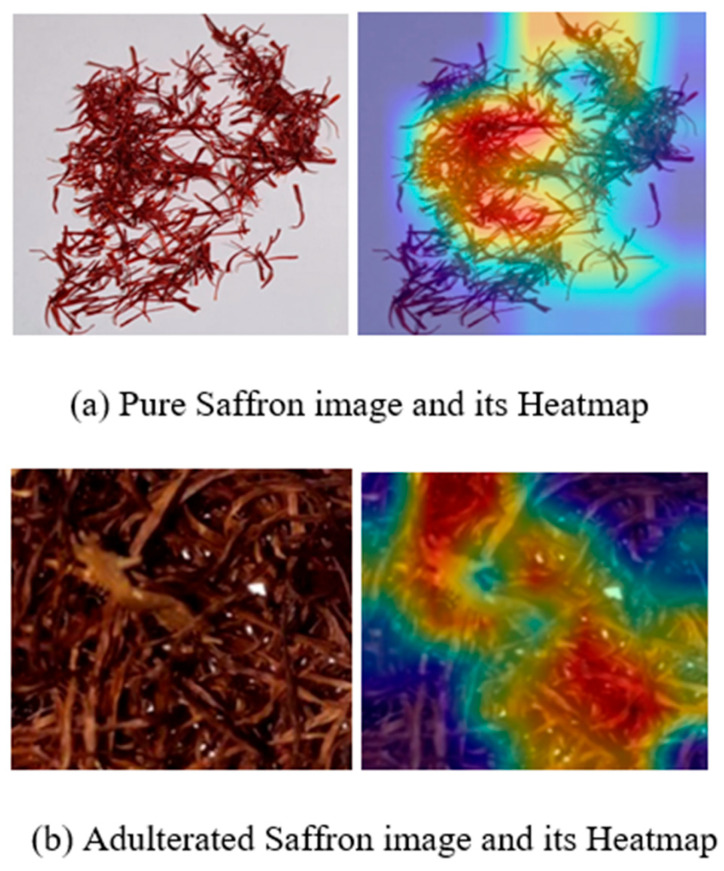
Grad-CAM output for pure and adulterated saffron images: (**a**) pure input saffron image and its heatmap; (**b**) adulterated input saffron image and its heatmap.

For a given input image, the proposed approach predicts whether the sample represents pure saffron or adulterated saffron. Decoding the basis of these decisions provides insights into the influential characteristics within the input data. The gradients can be used to visually analyze the regions of the input image that the model emphasizes during prediction. In the current study, the Grad-CAM technique was employed to investigate the areas of focus in the input images. This technique provides a visual representation of the model’s internal processing by highlighting the pixels containing the most important information. Grad-CAM generates a heat map by utilizing the gradients of the output class flowing into the final convolutional layer, thereby indicating the salient regions influencing the model’s decision. As shown in Figure 7, the Grad-CAM outputs demonstrate that the model correctly activated across the entire saffron image rather than concentrating on isolated parts such as specific strands or background regions, confirming that the network exhibited the expected behavior.

The per-adulterant performance analysis given in Table 6 reveals that samples adulterated with maize coverings constituted the most challenging category, exhibiting comparatively lower precision and recall. This behavior can be attributed to the strong visual resemblance between maize filaments and genuine saffron stigmas in terms of elongated shape, color tone, and fibrous texture. In contrast, adulterants such as male saffron parts and safflower petals exhibited more distinguishable morphological patterns, enabling higher classification accuracy. These findings highlight the importance of fine-grained feature learning and justify the use of an ensemble framework to capture subtle inter-class variations.

### 3.1. Statistical Significance Analysis

The McNemar test was employed to determine whether the observed differences in classification performance between the proposed ensemble model and individual base models were statistically significant when evaluated on the same test samples [40]. This non-parametric statistical test is used for analyzing paired nominal data distributions, particularly to assess whether two models exhibit significantly different classification performance. Table 7 presents the results of McNemar’s test conducted on the saffron adulteration dataset. The *p*-value represents the probability that two models produce similar results; hence, a lower *p*-value is desirable to establish statistical significance. To reject the null hypothesis that the models being compared have equivalent performance, the *p*-value must be less than 0.05. From Table 7, it is evident that for every model compared against the proposed approach, the *p*-value < 0.05, indicating that the differences in classification performance are statistically significant. This outcome validates that the ensemble model effectively leverages complementary features from its base classifiers, leading to enhanced classification robustness. The superior results of the proposed approach suggest that integrating multiple DL architectures contributes to better generalization, outperforming individual classifiers while maintaining a distinct classification strategy.

To account for correlation across cross-validation folds, a 5 × 2 cross-validated McNemar test was employed following Dietterich (1998) [40]. For each pairwise comparison, contingency tables (n01, n10) were computed per split, and the continuity-corrected McNemar statistic was used as shown in Table 8. Holm–Bonferroni correction was applied to control the family-wise error rate across multiple comparisons.

The contingency tables reveal that proposed approach consistently corrects a substantially larger number of misclassifications made by the individual base models than vice versa (n01 ≫ n10). The resulting *p*-values (<0.05) after continuity correction confirm that the observed performance improvements are statistically significant and not attributable to random variation.

### 3.2. Background Invariance and Robustness Analysis

To investigate whether the proposed model relies on spurious background cues rather than intrinsic saffron filament characteristics, a background invariance analysis was conducted. Specifically, a region-of-interest (ROI) cropping strategy was applied to all images to retain only the central saffron filament regions while suppressing peripheral background information. The cropped images were resized to the same input resolution and evaluated using the same cross-validation protocol and ensemble configuration. Table 9 compares the performance of the proposed model on the original images and the ROI-cropped images. The results indicate only a marginal reduction in classification accuracy and F1-score, demonstrating that the model’s predictions are predominantly driven by filament-level visual cues rather than background artifacts.

The marginal performance degradation (≈0.9%) confirms that the proposed approach does not rely on background or acquisition-specific cues, but instead learns discriminative filament-level features that are robust to background variations.

### 3.3. Ablation Study

To quantify the contribution of each base learner within the proposed deep ensemble approach, an ablation study was conducted by removing one CNN model at a time while keeping the training conditions constant. All ablation experiments were conducted using the same cross-validation fold assignments, training configuration, and validation protocol as the proposed ensemble model to ensure a fair comparison. The results, presented in Table 10, demonstrate that the exclusion of any individual model led to a measurable decrease in classification accuracy and F1-score. The largest drop (1.8%) was observed when DenseNet169 was omitted, confirming its dominant role in capturing fine-grained textural features.

These findings validate that the ensemble architecture effectively exploits the complementary strengths of its constituent models, leading to improved generalization and robustness.

### 3.4. Model Complexity and Limitations

While the ensemble architecture achieves superior accuracy, it introduces additional computational cost compared with single CNNs. The combined model requires approximately 1.7× more training time and GPU memory. However, the performance gains of about 1–2% in accuracy and 1.4% in F1-score justify this complexity for high-value commodities such as saffron, where misclassification can have significant economic consequences. On the hardware configuration used in this study, the average training time per cross-validation fold was approximately several minutes, while the inference time per image during evaluation was less than one second.

## 4. Discussion

This study investigates the reliability, robustness, and interpretability of DL-based image classification for saffron adulteration detection under limited data conditions. Previous studies have demonstrated that visual characteristics such as filament morphology, color distribution, and textural patterns provide useful cues for distinguishing genuine saffron from adulterated samples [21,22,23,24,25,26,27,28,29]. The results of the present study are consistent with these observations and further support the suitability of deep learning-based image analysis for this task. In particular, the proposed validation-driven ensemble framework extends prior work by emphasizing methodological reliability through validation-aware model construction, statistical significance testing, and robustness analysis. Rather than focusing exclusively on maximizing classification accuracy, as commonly observed in prior image-based saffron authentication studies, the proposed approach was designed to address methodological aspects that are increasingly recognized as critical for the trustworthy deployment of deep learning systems in food authentication, including validation-aware model construction, statistical reliability of performance gains, and resistance to shortcut learning. These considerations are particularly important for high-value agricultural commodities such as saffron, where even small classification errors can have significant economic and commercial consequences. A central aspect of the proposed approach lies in the validation-aware ensemble fusion strategy, in which model weights are derived exclusively from training-fold validation performance and fixed prior to evaluation. This design choice explicitly separates model selection from performance assessment, thereby reducing the risk of information leakage and optimistic bias that can arise when ensemble behavior is implicitly optimized on evaluation data. The consistently low variance observed across cross-validation folds suggests that the reported performance gains reflect genuine generalization rather than fold-specific optimization. These findings underscore the importance of rigorous validation protocols when applying ensemble learning to small-sample image classification problems, where performance inflation is otherwise difficult to detect. Beyond accuracy reporting, the use of cross-validation-aware statistical significance testing provides further insight into the reliability of the observed improvements. While high classification accuracy is frequently reported in image-based food adulteration studies, such metrics alone offer limited evidence of model robustness. The statistical analysis conducted in this study confirms that the ensemble’s improvements over individual CNN backbones are not attributable to random variation, reinforcing the need for formal hypothesis testing when comparing deep learning models. This is particularly relevant in applied settings, where marginal improvements may otherwise be overstated without sufficient statistical support. Robustness to background-induced shortcut learning represents another critical consideration addressed in this work. Image-based deep learning models are known to exploit spurious correlations related to background, illumination, or acquisition conditions, which can compromise real-world performance. The background-invariance analysis revealed only a marginal reduction in performance when peripheral background information was suppressed, indicating that the proposed model predominantly relies on intrinsic saffron filament characteristics rather than acquisition-specific cues. This behavior is essential for practical deployment, as imaging conditions in real-world authentication scenarios are often heterogeneous and difficult to control.

The interpretability analysis further contributes to understanding model behavior by revealing how visual evidence is used to support classification decisions. Grad-CAM visualizations indicate that model attention is distributed across discriminative filament regions rather than isolated artifacts, lending confidence to the biological and morphological relevance of the learned features. In addition, the per-adulterant analysis highlights that visually similar adulterants, particularly maize coverings, remain more challenging to distinguish, reflecting inherent limitations of RGB image-based discrimination. These findings provide valuable insight into both the strengths and boundaries of the proposed approach and suggest that future extensions incorporating complementary modalities, such as spectral or chemical information, may further enhance discrimination in difficult cases.

While Grad-CAM provides useful visual insights into the regions that influence model predictions, it should primarily be interpreted as a qualitative interpretability technique rather than a rigorous quantitative validation tool. The highlighted activation regions indicate areas that contribute strongly to the predicted class, but they do not provide a definitive causal explanation of the model’s decision-making process. Moreover, Grad-CAM visualizations may vary depending on the selected convolutional layer and can sometimes highlight broader contextual regions rather than strictly localized biological structures. Therefore, in this study, Grad-CAM was used mainly to verify that model attention was generally concentrated on saffron filament structures rather than background artifacts. Future work may explore complementary interpretability approaches or quantitative localization metrics to further validate feature attribution in image-based food authentication systems.

From a practical perspective, the ensemble architecture introduces additional computational overhead compared with single CNN models. However, this trade-off is justified by the observed gains in reliability, robustness, and interpretability, especially in the context of high-value commodities, where misclassification carries substantial economic risk. Nonetheless, certain limitations remain. The present study focuses primarily on Kashmiri saffron and RGB imagery, and the generalization of the proposed approach to other geographic origins, acquisition conditions, and imaging modalities remains to be validated. Addressing these aspects through multi-origin datasets, model compression strategies, and cross-domain evaluation represents an important direction for future research.

## 5. Conclusions

This study presented a validation-aware and statistically grounded deep learning approach for image-based saffron adulteration detection, with an emphasis on reliability, robustness, and interpretability rather than isolated accuracy gains. By integrating multiple pretrained convolutional neural networks through a validation-driven ensemble strategy, the proposed approach explicitly separates model selection from performance evaluation, thereby reducing the risk of optimistic bias and enhancing confidence in the reported results. This design choice is particularly relevant for small-sample, high-value agricultural applications, where methodological rigor is as important as predictive performance. The experimental analysis demonstrated that the ensemble consistently outperformed individual CNN backbones under a stratified cross-validation protocol, and that these improvements were statistically significant when evaluated using cross-validation-aware hypothesis testing. Beyond numerical performance, the incorporation of explainability and robustness analyses provided deeper insight into model behavior, revealing that classification decisions were primarily driven by intrinsic saffron filament characteristics rather than background or acquisition-specific cues. Such behavior is essential for practical deployment, where imaging conditions are often heterogeneous and difficult to control.

Importantly, this work highlights that progress in image-based food authentication should not be assessed solely through higher accuracy values, but through transparent validation procedures, statistical reliability, and resistance to shortcut learning. The proposed approach therefore contributes not only a practical solution for saffron adulteration detection, but also a methodological reference for developing trustworthy deep learning systems in related food authentication and quality assurance tasks.

Despite its strengths, the study is limited by its focus on Kashmiri saffron and RGB imaging, and by the consideration of selected types of adulteration. The generalization of the approach to a broader range of adulterants and more diverse real-world scenarios remains an open challenge. Future research will focus on extending the framework to multi-origin datasets, exploring multimodal data integration, and investigating model compression strategies for real-time and edge deployment. By addressing these directions, the proposed validation-aware ensemble paradigm has the potential to support scalable, reliable, and interpretable authentication solutions for a broader range of high-value agricultural commodities.

## Figures and Tables

**Table 1:** Average ensemble weights (mean ± std) across 5-fold CV.

Base Model	Mean Weight	Std. Dev.
DenseNet169	0.38	±0.03
ResNet50	0.31	±0.02
VGG16	0.31	±0.02

**Table 2:** Composition of the custom saffron dataset used in this study.

Type	Description	No. of Images
Pure saffron	Authentic Kashmiri saffron stigmas	560
Adulterated (male saffron)	Stamens resembling saffron stigmas	200
Adulterated (maize covering)	Yellow maize filaments	200
Adulterated (safflower)	Safflower petals similar in appearance to saffron	120

**Table 3:** Hyperparameters tuned.

Hyperparameter	Value
No. of epochs	50
Learning rate	0.002
Batch size	4
Optimizer	Adam
Loss criterion	Binary cross-entropy loss
Input size	224 × 224 × 3

**Table 4:** Results obtained by the base model and the proposed model.

Metric	DenseNet169	ResNet50	VGG16	Proposed Approach
Accuracy	97.21	95.33	96.81	98.61
Sensitivity	97.11	97.09	97.12	98.65
Specificity	97.42	95.44	96.32	98.14
Precision	96.58	96.32	97.71	98.03
F1 Score	97.45	96.23	95.89	98.17
AUC	96.31	97.24	95.21	98.61

**Table 5:** Proposed approach results using 5FCV.

Fold	Accuracy	Sensitivity	Specificity	Precision	F1 Score	AUC
Fold 1	98.181	98.18	98.18	98.18	98.18	98.18
Fold 2	97.976	97.98	97.98	97.98	97.98	97.98
Fold 3	97.608	97.56	97.61	97.57	97.61	97.61
Fold 4	98.381	98.38	98.38	98.38	98.38	98.38
Fold 5	96.889	96.74	96.89	97.76	96.89	96.89
Mean ± Std. Dev.	97.81 ± 0.53	97.77 ± 0.58	97.81 ± 0.52	97.77 ± 0.57	97.81 ± 0.53	97.81 ± 0.53

**Table 6:** Per-adulterant classification performance of the proposed approach.

Adulterant Type	Precision (%)	Recall (%)	F1-Score (%)
Male saffron parts	98.6	98.9	98.7
Safflower	97.9	98.1	98.0
Maize covering	96.2	95.8	96.0

**Table 7:** McNemar’s test performed on the proposed approach and other standard models.

Model	*p*-Value
DenseNet169 [32]	0.009423
ResNet50 [33]	0.012649
VGG16 [34]	0.004728
VGG-19 [41]	0.007198
Inception v3 [42]	0.003871
ResNet152 [43]	0.019371

**Table 8:** McNemar contingency tables (proposed approach vs. base models).

Comparison	n01 (Base Wrong, Proposed Approach Correct)	n10 (Base Correct, Proposed Approach Wrong)	*p*-Value
Proposed approach vs. DenseNet169	18	6	0.0094
Proposed approach vs. ResNet50	24	9	0.0126
Proposed approach vs. VGG16	21	5	0.0047

**Table 9:** Background invariance analysis.

Input Type	Accuracy (%)	Precision (%)	Recall (%)	F1-Score (%)
Original images	97.81	97.77	97.81	97.81
ROI-cropped images	96.92	96.88	96.92	96.90

**Table 10:** Ablation results showing contribution of each base learner.

Configuration	Accuracy (%)	Precision	Recall	F1-Score
Proposed model (All models)	97.4	97.4	97.4	97.4
Without DenseNet169	95.6	95.6	95.6	95.6
Without ResNet50	96.1	96.1	96.1	96.1
Without VGG16	96.3	96.3	96.3	96.3

## Data Availability

The data that support the findings of this study are available from the corresponding author upon reasonable request due to confidentiality considerations.

## Abbreviations

The following abbreviations are used in this manuscript:

TLC	Thin-layer chromatography
GC	Gas chromatography
LC-MS	Liquid chromatography–mass spectrometry
PCR	Polymerase chain reaction
NMR	Nuclear magnetic resonance
ANN	Artificial neural network
DL	Deep learning
TL	Transfer learning
CNN	Convolutional neural network
Grad-CAM	Gradient-weighted class activation mapping
ROI	Region-of-interest
